# Does government health insurance protect households from out of pocket expenditure and distress financing for caesarean and non-caesarean institutional deliveries in India? Findings from the national family health survey (2019-21)

**DOI:** 10.1186/s13104-023-06335-w

**Published:** 2023-05-22

**Authors:** Samir Garg, Narayan Tripathi, Kirtti Kumar Bebarta

**Affiliations:** State Health Resource Centre, Chhattisgarh, Raipur India

**Keywords:** Institutional deliveries, Caesarean, PMJAY, Ayushman Bharat, Pradhan Mantri Jan Arogya Yojana, Out of pocket expenditure, Distress financing, India, Health Insurance, Publicly Funded Health Insurance, Financial Protection, Private sector

## Abstract

**Objective:**

Institutional deliveries have been promoted in India to reduce maternal and neonatal mortality. While the institutional deliveries have increased, they tend to involve large out of pocket expenditure (OOPE) and distress financing for households. In order to protect the families from financial hardship, publicly funded health insurance (PFHI) schemes have been implemented in India. An expanded national health insurance scheme called the Ayushman Bharat Pradhan Mantri Jan Arogya Yojana (PMJAY) was launched in 2018. The current study was aimed at evaluating the performance of PFHI in reducing the OOPE and distress financing for the caesarean and non-caesarean institutional deliveries after the launch of PMJAY. This study analysed the nationally representative dataset of the National Family Health Survey (NFHS-5) conducted in 2019-21.

**Results:**

Enrollment under PMJAY or other PFHI was not associated with any reduction in out of pocket expenditure or distress financing for caesarean or non-caesarean institutional deliveries across India. Irrespective of the PFHI coverage, the average OOPE in private hospitals was five times larger than public hospitals. Private hospitals showed an excessive rate of using caesarean-section. Utilization of private hospitals was significantly associated with incurring larger OOPE and occurrence of distress financing.

**Supplementary Information:**

The online version contains supplementary material available at 10.1186/s13104-023-06335-w.

## Background

The Sustainable Development Goals (SDGs) agreed by countries across the globe include targets of reducing the maternal deaths to 70 per 100,000 live births and the neonatal deaths to 12 per 1000 live births by 2030 [1]. Most of the low- and middle-income countries (LMICs) including India have implemented the strategy of promoting institutional deliveries to ensure the necessary maternal and neonatal care [2, 3]. On the supply side, the national health mission in India had expanded the availability of services for institutional deliveries including the emergency obstetric care under the Janani Shishu Suraksha Karyakram [4]. Additionally, a cash-transfer scheme known as the Janani Suraksha Yojana (JSY) was introduced in 2005 to encourage institutional deliveries [5].

There is no doubt that institutional deliveries including the caesarean deliveries have increased sharply in India [4, 5]. The maternal mortality ratio of India has come down from 254 in 2004-06 to 103 per 100,000 live births in 2017-19 [4, 6]. The neonatal mortality declined from 38 to 20 per 1000 live births between 2005 and 2019 [7]. However, there has been a concern regarding the large out of pocket expenditure (OOPE) households face for institutional deliveries [8–10]. India has a mixed health system with a sizeable presence of private hospitals. Studies have reported significant financial distress for institutional deliveries and the situation was worse in case of caesarean deliveries [8–14]. Incurring a large OOPE can force families to arrange the funds in ways that can have further adverse consequences for them [8]. This is known as distress financing and it usually involves borrowing money or selling family’s assets [8].

In order to protect households from financial hardship, governments in India have implemented publicly funded health insurance (PFHI) schemes [15, 16]. A national PFHI scheme known as the Rashtriya Swasthya Bima Yojana (RSBY) was initiated in 2008 [16]. Multiple studies showed that the RSBY scheme was ineffective in ensuring financial protection [16–19]. In 2018, RSBY was replaced by a larger national scheme known as the Ayushman Bharat - Pradhan Mantri Jan Arogya Yojana (PMJAY) [20]. PMJAY covers 100 million poor households with an annual sum of half a million Indian Rupees (around 7000 USD) per household for inpatient care [20]. The states have added resources to further expand the population coverage under PMJAY. The services under PMJAY are expected to be completely free for the enrolled persons and ‘cash-less’ at the point of care [21]. Under PMJAY, states empanel a mix of private and public hospitals to provide in-patient services at pre-defined prices.

The existing studies on institutional deliveries in India have not focused on examining the effectiveness of PFHI in reducing OOPE. Further, none of the studies have examined the financial protection for institutional deliveries after the launch of PMJAY. The fifth wave of the National Family Health Survey (NFHS-5) was conducted in 2019-21 and the dataset has become available very recently [22]. The current study was aimed at answering the question – Was PFHI effective in reducing OOPE and distress financing for the caesarean and non-caesarean institutional deliveries in India.

## Materials and methods

### Dataset

This study used the dataset of the NFHS-5 survey [22]. This household survey took place in two phases from June 2019 to April 2021 and collected data from 636,699 households. The government survey had a nationally representative sample with a stratified two-stage sampling design. The detailed sampling design of NFHS-5 can be found in its official report [22].

For child-birth, NFHS-5 used a recall period of five years, covering 201,311 institutional deliveries. For the purpose of the current study, the recall period was taken as one year i.e., its sample included only those deliveries that took place within a year of the survey. This was done to reduce the recall bias and to allow the study on focus on the period 2018 onwards. For a Type-1 error of 5%, power of 95% and a design effect of 1.5 to account for multi-stage sampling; a sample size of 572 institutional deliveries was required. The current study analyzed 42,978 institutional deliveries including 10,427 caesarean deliveries and the sample size was sufficient for the required analyses.

### Statistical analysis

The list of study variables is given in Additional file S1. The data was analyzed using STATA-15. Descriptive analyses were conducted using cross-tabulations. Confidence intervals (CI) were reported at 95%. Out of Pocket Expenditure (OOPE) was calculated for each episode by adding the medical expenses (inpatient services, medicines, diagnostics) and expenditure on emergency transportation and deducting any prepayments made or cash reimbursements received by the household. The logarithmic transformation of OOPE was used for the multivariate analyses as it offers advantages in addressing any skew or extreme values in OOPE [15, 21]. Distress financing was defined as borrowing of money or selling of assets by households to meet the OOPE [8].

Ordinary least squares (OLS) regression models were applied for the log of OOPE and OOPE. A logistic regression model was applied to find out the determinants of distress financing incidence. For robustness, propensity score matching (PSM) was used to examine the effect of PFHI-enrolment on OOPE and distress financing [16, 21]. PSM reduced the confounding by creating a matched sample of the treated (PFHI-enrolled) and untreated (not-enrolled) participants.

The above analyses were done for all institutional deliveries first and then repeated for the caesarean and non-caesarean institutional deliveries. Significance was taken at 95% (p < 0.05).

## Results

The sample profile is given in Additional file S2. Around 82% of the sample institutional deliveries belonged to years 2019 and 2020. Around 26% of the women delivering in institutions were covered under PFHI. Among the institutional deliveries, 24.2% involved caesarean-section.

### Type of hospital utilised

Around 74% of all institutional deliveries took place in public hospitals. The public facilities accounted for 47.2% of the caesarean deliveries (Table 1).

**Table 1:** PFHI coverage and share of public and private facilities in institutional child births with 95% CI

Overall institutional child births	Public facility (%)	Private facility (%)
	n = 31,848	n = 11,130
All	74.1 (73.7–74.5)	25.9 (25.5–26.3)
PFHI-enrolled	76.58 (75.8–77.3)	23.4 (22.6–24.2)
Not insured	73.2 (72.7–73.7)	26.7 (26.2–27.2)
**Caesarean deliveries**	47.2 (46.3–48.2)	52.8 (51.8–53.7)
PFHI-enrolled	48.7 (46.8–50.5)	51.3 (49.4–53.2)
Not insured	46.7 (45.6–47.8)	53.2 (52.1–54.4)
**Non-caesarean deliveries**	82.74 (82.3–83.1)	17.2 (16.8–17.7)
PFHI-enrolled	85.3 (84.5–86)	14.7 (13.9–15.4)
Not insured	81.8 (81.3–82.3)	18.1 (17.6–18.6)

### Caesarean-section rate

The proportion of caesarean deliveries in the total institutional deliveries was 15.5% (15.1-15.9%) for public hospitals and 49.5% (48.6-50.5%) for private hospitals.

### OOPE

The caesarean deliveries were more expensive than the non-caesarean ones (Table 2). The private hospitals were more expensive than public facilities. The mean OOPE for caesarean deliveries in private sector was around five times the amount in public sector. A similar comparison was found for the mean OOPE for non-caesarean deliveries in private and public facilities. The mean OOPE for those enrolled under PFHI was similar to the uninsured. A similar pattern was visible when the median OOPE was compared instead of the mean (Table 2).

**Table 2:** Mean and Median OOPE for per institutional delivery (in INR) with 95% CI ( ) among the PFHI-enrolled and not-enrolled

**a. Mean OOPE for per institutional child births (in INR) with 95% CI**			
**Particulars**	**All Institutional deliveries (N = 42,978)**	**Caesarean deliveries (N = 10,427)**	**Non-caesarean deliveries (N = 32,551)**
Public overall	2541 (2457–2624)	5593 (5288–5897)	1985 (1905–2065)
Public for PFHI-enrolled	2235 (2100–2370)	5236 (4541–5933)	1705 (1609–1801)
Public without insurance	2653 (2550–2753)	5719 (5389–6049)	2089 (1985–2192)
Private overall	18,163 (17,736–18,590)	25,956 (24,575–26,015)	11,241 (10,851–11,631)
Private for PFHI-enrolled	17,627 (16,732–18,521)	24,205 (22,740–25,670)	10,526 (9716–11,337)
Private without insurance	18,327 (17,841–18,813)	25,652 (24,825–26,479)	11,445 (11,000–11,880)
**b. Median OOPE for per institutional child births (in INR) with 95% CI**			
**Particulars**	**All Institutional deliveries (N = 42,978)**	**Caesarean deliveries (N = 10,427)**	**Non-caesarean deliveries (N = 32,551)**
Public overall	700 (700–800)	2000 (2000–2200)	500 (500–550)
Public for PFHI-enrolled	500 (500–500)	2000 (1500–2000)	500 (303–500)
Public without insurance	900 (800–1000)	2111 (2000–2500)	700 (600–700)
Private overall	11,000 (10,792–11,507)	20,033 (20,000–21,000)	8000 (7000–8000)
Private for PFHI-enrolled	10,500 (10,000–11,500)	19,300 (17,500–20,000)	7000 (6000–8000)
Private without insurance	11,033 (11,000–12,000)	21,000 (20,033–22,000)	8000 (8000–8400)

### Effect of PFHI enrollment on the size of OOPE

The OLS model for log of OOPE for institutional deliveries showed no significant association between PFHI-enrollment and the size of OOPE (Additional file S3). Utilisation of the private facilities for institutional deliveries was associated significantly with greater OOPE. The OLS model for OOPE also showed similar results (Additional files S3).

The above OLS models were repeated for caesarean deliveries alone and the pattern of results remained same (Additional files S4). The above models when repeated for non-caesarean deliveries also showed similar results (Additional files S5).

The PSM models for log of OOPE or OOPE on institutional deliveries showed that the PFHI-enrolment did not have any effect (Additional File S6). The results did not change when PSM models were applied for the caesarean and non-caesarean deliveries separately (Additional File S6).

### Distress financing

Overall, 26.7% of the caesarean deliveries and 19.2% of the non-caesarean deliveries involved distress financing. The incidence of distress financing was greater for utilizing private facilities as compared to public facilities (Table 3). There was hardly any difference in the distress financing between the PFHI-enrolled and the uninsured. (Table 3).

**Table 3:** Incidence of Distress financing (%) in institutional deliveries with 95% CI

Particulars	Overall Institutional child births (N = 42,978)	Caesarean deliveries (N = 10,427)	Non-caesarean deliveries (N = 32,551)
Overall	21.2 (20.8–21.7)	26.7 (25.8–27.6)	19.2 (18.8–19.6)
Public overall	18.7 (18.2–19.2)	20.6 (19.4–21.9)	18.3 (17.8–18.8)
Public for PFHI-enrolled	18.25 (17.3–19.2)	21.3 (18.9–23.8)	17.5 (16.7–18.7)
Public without insurance	18.9 (18.3–19.4)	20.4 (19.0-21.8)	18.6 (17.9–19.2)
Private overall	27.3 (26.4–28.2)	31.5 (30.3–32.8)	23.1 (22.0-24.3)
Private for PFHI-enrolled	26.9 (25.2–28.8)	31.2 (28.7–33.8)	22.2 (20.0-24.8)
Private without insurance	27.4 (26.4–28.4)	31.65 (30.2–33.1)	23.3 (22.1–24.7)

### Effect of PFHI enrollment on distress financing

The multivariate logistic regression model showed that enrollment under PFHI was not associated with reduced incidence of distress financing. Utilizing private facilities, caesarean delivery and duration of hospitalization were the independent predictors of distress financing (Additional file S7). The results did not change when the above logistic models were applied separately for the caesarean and the non-caesarean deliveries (Additional File S7).

The PSM model showed that PFHI-enrolment did not have any effect on the incidence of distress financing for institutional deliveries (Additional file S6). The results did not change when the above PSM models were applied separately for the caesarean and the non-caesarean deliveries (Additional file S6).

## Discussion

The PMJAY, launched in 2018, is a flagship policy of Indian government for financial protection in healthcare. The current study is the first to examine the effectiveness of PFHI in financial protection for institutional deliveries after the above important policy got implemented. The current study used a household survey with a nationally representative sample for India. PMJAY covered caesarean deliveries in all states and non-caesarean deliveries in some states during the period covered by the present study.

The existing studies had shown that households incur significant OOPE for institutional deliveries including emergency obstetric care [8–14]. A study based on the NFHS dataset of 2015-16 showed that distress financing was higher among the poor and those utilising private facilities [8]. Another study was based on the National Sample Survey (NSS) dataset of 2014 and it showed that health insurance schemes could not prevent catastrophic health expenditure [10]. The current study showed that the above problem has persisted in 2019-21 period when PMJAY was in operation.

In India, NSS is the most commonly used source of national data on healthcare OOPE. The NFHS is the key national dataset in India for the sexual and reproductive health and it can also be an important source for data on OOPE for institutional deliveries. The results of the current study along with the existing literature on PFHI in India show that such schemes are not effective in reducing OOPE or protecting people from financial distress. The above finding has not changed when the data from different national or state level household surveys was analysed by various Indian studies [15, 16, 21, 23]. The profit expectations of the private sector providers are very high in India and they are poorly regulated [24, 25]. The ineffectiveness of PFHI could be due to double-billing and overcharging by private providers and the failure of contracting in regulating provider behaviour [15, 18, 21, 26].

The use of private facilities continued to involve large OOPE for those enrolled under PMJAY and other PFHI schemes in the 2019-21 period. Irrespective of PFHI coverage, private hospitals were several times more expensive than the public facilities in the current study. This is also a recurring finding in studies of Indian health system [21, 23]. The excessive rate of using caesarean-section in private hospitals is another long standing concern [27, 28]. It indicates the problem of supply induced utilisation under the for-profit healthcare [28, 29].

The current study found that the public sector had gained a majority share in institutional deliveries and covered almost half of the caesarean deliveries too. The average rate of caesarean section in public sector was neither inadequate nor excessive. The OOPE in public facilities was at least five times lower than the private facilities. The national health mission of India had focused on strengthening the public sector to deliver maternal care services and the strategy seems to be paying off [4, 30].

India has used PFHI based policies for more than fifteen years now and studies based on multiple household surveys show that they have failed in achieving their fundamental purpose of ensuring financial protection. It suggests the need to devise alternative policies to realize the vision of UHC and SDGs.

### Limitations

The study is cross-sectional. Around 5.1% of the sample belonged to the period before the start of PMJAY. The quality of services can influence OOPE but the current study could not take it into account.

## Electronic supplementary material

Below is the link to the electronic supplementary material.

Supplementary Material 1

Supplementary Material 2

Supplementary Material 3

Supplementary Material 4

Supplementary Material 5

Supplementary Material 6

Supplementary Material 7

## Data Availability

The dataset used and analyzed during the current study is available in public domain. It is freely available for research and academic purposes. It can be downloaded at https://www.dhsprogram.com/data/dataset/India_Standard-DHS_2020.cfm?flag=0.

## Abbreviations

CI: Confidence Interval
INR: Indian Rupees
LMICs: Low- and Middle-Income Countries
NFHS: National Family Health Survey
NSS: National Sample Survey
OOPE: Out-of-pocket expenditure
PFHI: Publicly Funded Health Insurance
PMJAY: Pradhan Mantri Jan Arogya Yojana
USD: US dollars
